# Validity of the estimated angular information obtained using an inertial motion capture system during standing trunk forward and backward bending

**DOI:** 10.1186/s13102-024-00942-1

**Published:** 2024-07-17

**Authors:** Taiki Morikawa, Nariyuki Mura, Toshiaki Sato, Hiroshi Katoh

**Affiliations:** 1 Department of Rehabilitation, Eniwa Hospital, Eniwa, Hokkaido 061-1449 Japan; 2 Graduate School, Yamagata Prefectural University of Health Sciences, Yamagata, Yamagata 990-2212 Japan

**Keywords:** Reproducibility of results, Spine, Wearable electronic devices, Motion capture

## Abstract

**Background:**

Bending the trunk forward and backward while standing are common daily activities and can have various patterns. However, any dysfunction in these movements can considerably affect daily living activities. Consequently, a comprehensive evaluation of spinal motion during these activities and precise identification of any movement abnormalities are important to facilitate an effective rehabilitation. In recent years, with the development of measurement technology, the evaluation of movement patterns using an inertial motion capture system (motion sensor) has become easy. However, the accuracy of estimated angular information obtained via motion sensor measurements can be affected by angular velocity. This study aimed to compare the validity of estimated angular information obtained by assessing standing trunk forward and backward bending at different movement speeds using a motion sensor with a three-dimensional motion analysis system.

**Methods:**

The current study included 12 healthy older men. A three-dimensional motion analysis system and a motion sensor were used for measurement. The participants performed standing trunk forward and backward bending at comfortable and maximum speeds, and five sensors were attached to their spine. Statistical analysis was performed using the paired t-test, intraclass correlation coefficient, mean absolute error, and multiple correlation coefficient.

**Results:**

Results showed that the estimated angular information obtained using each motion sensor was not affected by angular velocity and had a high validity.

**Conclusions:**

Therefore, the angular velocity in this study can be applied clinically for an objective evaluation in rehabilitation.

**Supplementary Information:**

The online version contains supplementary material available at 10.1186/s13102-024-00942-1.

## Background

Standing with the trunk bending forward (trunk forward bending) and bending backward (trunk backward bending) are common daily living activities that may have various patterns. When these movements become impaired, they can considerably affect activities of daily living. Therefore, a detailed assessment of spinal movements during standing with trunk forward and backward bending as well as an accurate understanding of any movement disorders is essential to facilitate effective rehabilitation [1–4]. Generally, to determine the range of motion of the thoracic and lumbar spine from the start to the end of the task movement and the distance traveled, trunk forward and backward bending can be measured using goniometers and inclinometers [5], a kyphometer [6], and tape measures [7]. However, movement patterns from time-series data such as lumbar pelvic rhythm cannot be evaluated [8–11].

A markerless motion capture system measures movements and evaluates movement patterns in a time-series in clinical settings [12]. Nakano et al. [13] simultaneously measured the motions of the shoulder, elbow, hand, hip, knee, and ankle joints during walking, jumping, and throwing motions using a markerless system and an optical motion capture system (optical system) to verify the validity of the markerless system. Results showed that the measurement error of the markerless system was < 30 mm. Similarly, Vilas-Boas et al. [14] assessed the validity of spine motion in walking using a markerless system. They found that the error range of the markerless system was significant at 5°–33° and that the reliability and validity of the system were low. Recent studies have evaluated thoracic and lumbar spine kyphosis using Kinect [12], a commonly used markerless system [15, 16]. Results reveal that the system had a high intra- and interexaminer reliability. However, these studies only assessed static standing alignment and did not perform dynamic assessment of the spine.

In recent years, inertial motion capture systems (motion sensors) have attracted attention [1]. Compared with optical systems, motion sensors are inexpensive, lightweight, and compact, and they are suitable for clinical use because they can be used outdoors and have fewer restrictions. However, most conventional motion sensors can measure acceleration [17–21] and angular velocity [22–25]. The angle can be calculated by integrating angular rate data. However, drift occurs because of accumulated signal noise in the raw data [22, 26, 27]. To address this issue, a motion sensor with a built-in advanced arithmetic processor that calculates quaternions from three-axis acceleration and three-axis angular velocity data and that estimates angles has been developed [28]. Hence, the time-series angles can be accurately measured during motion.

The motion sensor by LEOMO was developed to improve its measurement performance in sport activities such as cycling and running. This was achieved by measuring the lower extremity joint angles using unique indices known as motion performance indicators. In terms of features, it provides highly reliable angle measurements in the sagittal plane [28] and can display angles in real-time.

A systematic review [29] showed that motion sensors had high reliability (ICC: 0.99) and validity, with a root mean square error of 2.9°. However, the accuracy of the estimated angle data obtained using motion sensors may be influenced by movement speed [30, 31]. Hence, verification must consider movement speed. To date, no studies have examined the effect of movement speed on trunk forward and backward bending.

Furthermore, in clinical practice, as trunk forward and backward bending are performed using various patterns and at different speeds during activities of daily living, their range must be validated. Verification should encompass the range from an individual’s comfortable speed to their maximum speed.

Therefore, this study aimed to measure trunk forward and backward bending at different movement speeds using motion sensors. Further, the validity of estimated angle information obtained from these measurements with a three-dimensional motion analysis system was compared.

## Methods

### Participants

This study recruited 12 healthy older men with the following characteristics: age: 66.3 ± 2.6 years, height: 166.9 ± 5.5 cm, weight: 64.6 ± 11.7, body mass index: 23.2 ± 4.4 kg/m^2^. All participants were registered with the Yamagata Prefecture Silver Human Resources Center. However, participants diagnosed with neurological or orthopedic disease within the past year and those with hyperkyphosis were excluded from the current analysis. This study was approved by the Eniwa Hospital Ethics Committee (no. 195), and a written informed consent was obtained from all participants after the study purpose was explained to them.

### Tasks

The task movements comprised trunk forward and backward bending, which were performed at two different speeds (comfortable speed and maximum speed) for each task under four conditions. The maximum speed was defined as the highest speed achievable by each participant. Both trunk forward and backward bending were performed with the knee joint set at 0° of extension.

### Measurement procedures

**Fig. 1:**
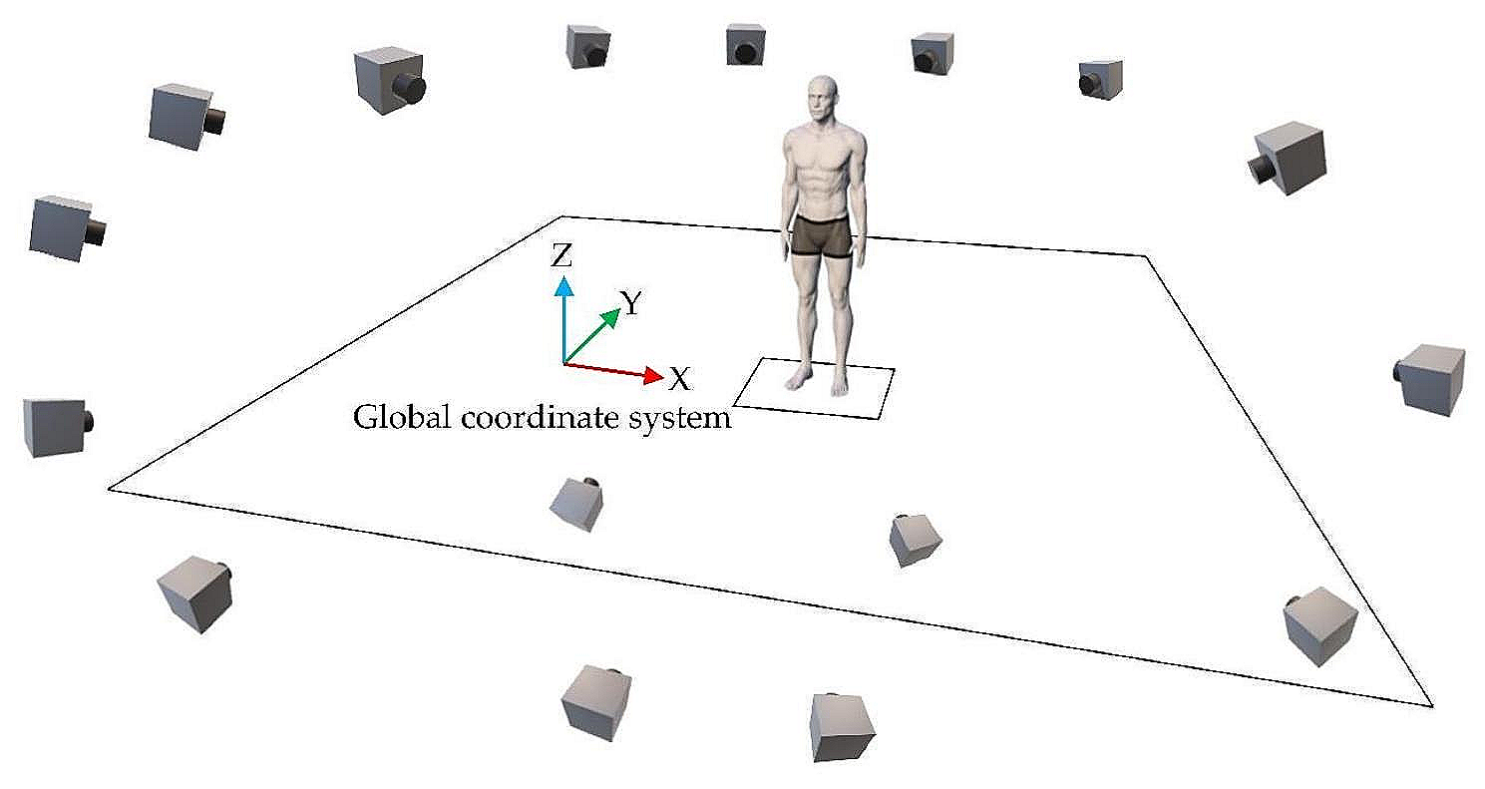
Measurement environment

**Fig. 2:**
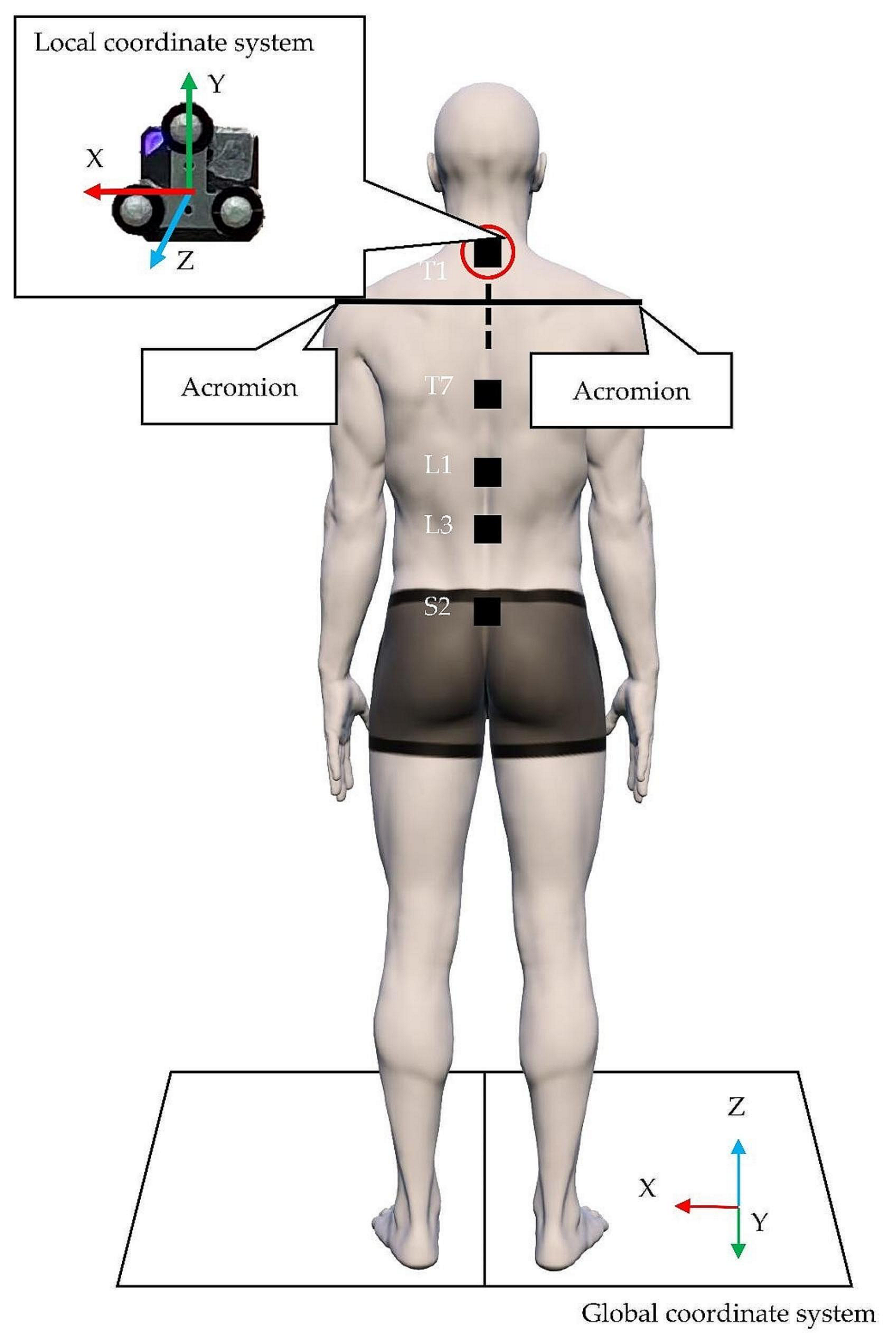
Placement of the motion sensor

For all measurements, this study used a three-dimensional motion analysis system (3DMA system), such as VICON MX16 (Vicon Motion Systems Ltd., Oxford, UK), with 16 cameras (Fig. 1) and five motion sensors (LEOMO, Tokyo, Japan) (dimensions: 37 mm [W] × 37 mm [D] × 7.8 mm [H], weight: 12 g), and five T-shaped stainless-steel plates (48 mm [W] x 40 mm [H]) (Fig. 2). The motion sensor by LEOMO had highly reliable angle measurements specialized for the sagittal plane [28].

The motion sensors were set by fixing the T-shaped stainless-steel plates with double-sided tapes. Thus, they aligned with the local coordinate X-axis and Y-axis of the motion sensors. Infrared markers were then attached to three points on the tip of each T-shaped stainless-steel plate. The Euler angles obtained from the 3DMA system were calculated using the processing software Nexus 2.12.0 (Vicon Motion Systems Ltd., Oxford, UK).

First thoracic spinous process: the spinous process one level below the maximum prominence when the neck is flexed.

Seventh thoracic spinous process: the midpoint of the line connecting the inferior angles of both scapulae.

First lumbar spinous process: the spinous process two levels above the L3 spinous process.

Third lumbar spinous process: the spinous process one level above the L4 spinous process, identified from the midpoint of the Jacoby’s line (L4/L5 interval).

Second sacrum spinous process: the midpoint of the line connecting both posterior superior iliac spines.

#### Trunk forward bending

The motion sensor was positioned to ensure proper alignment. The black line connecting the participant’s bilateral acromion was aligned with the X-axis of the local sensor’s coordinates. In addition, the Y-axis of the local sensor’s coordinates was aligned with a black line perpendicular to the black dotted line (Fig. 2). To attach the motion sensors, a double-sided tape was used and affixed to the first thoracic spinous process (T1), seventh thoracic spinous process (T7), first lumbar spinous process (L1), third lumbar spinous process (L3), and second sacrum spinous process (S2).

**Fig. 3:**
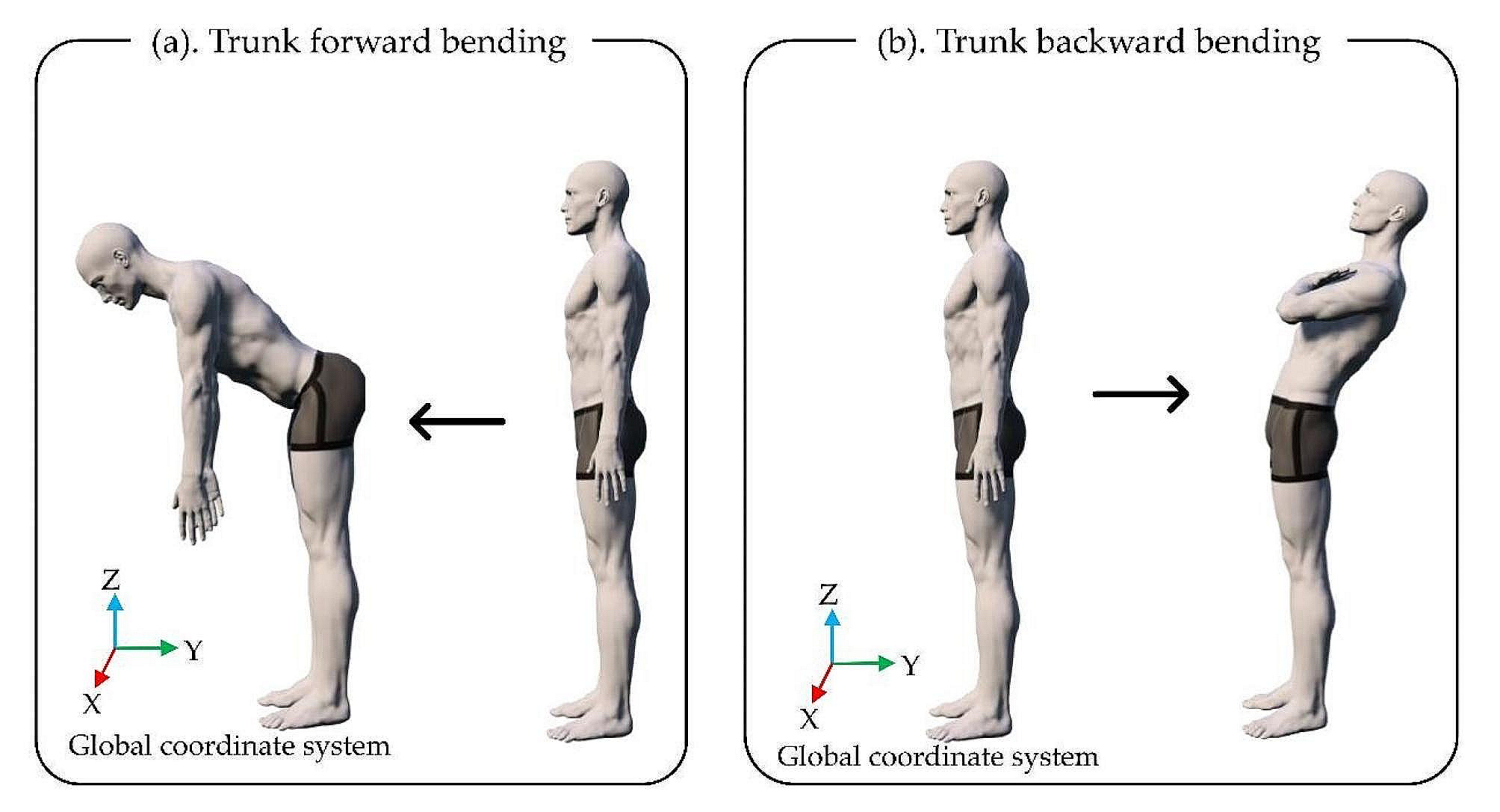
Trunk forward and backward bending. (**a**) Trunk forward bending. (**b**) Trunk backward bending

The participants were instructed to begin the activity by assuming a standing position with their feet placed shoulder-width apart and to bend their trunk forward until their fingertips touched their toes upon receiving a cue. After bending their trunk forward, they were instructed to return to the starting position. During trunk forward bending, measurements were conducted three times each at comfortable and maximum speeds, with each measurement comprising three consecutive repetitions (Fig. 3-a).

#### Trunk backward bending

The attachment of the motion sensor was similar to that in trunk forward bending.

The participants were required to begin the activity by assuming the standing position with their feet placed shoulder-width apart and crossing their arms on their chest to bend their trunk backward to the greatest extent possible upon receiving a cue. After bending their trunk backward, they were instructed to return to their starting position. During trunk backward bending, measurements were conducted three times each at comfortable and maximum speeds, with each measurement comprising three consecutive repetitions (Fig. 3-b).

### Data analysis

The analyzed parameters encompassed angle and angular velocity, range of motion (ROM) in trunk forward and backward bending, and waveform. The Euler angles rotated in the order of the XYZ axes using the 3DMA system were used. The estimated angles were calculated from the quaternion derived from the motion sensor angle (MS angle) [28]. The angle definition was 0° if the motion sensor was perpendicular to the floor, with negative values indicating trunk forward bending and positive values denoting trunk backward bending. Angular velocity was the maximum value obtained from the motion sensor during trunk forward and backward bending. ROM was defined as the amount of change from 0° at the initiation of movement to the maximum angle. The waveform was defined as the time-series data from the start to the end of the task. The sampling frequency was 100 Hz for the 3DMA system and the motion sensor. Data analysis was conducted after low-pass filtering (Butterworth filter) at 6 Hz.

#### Analysis of trunk forward bending

**Fig. 4:**
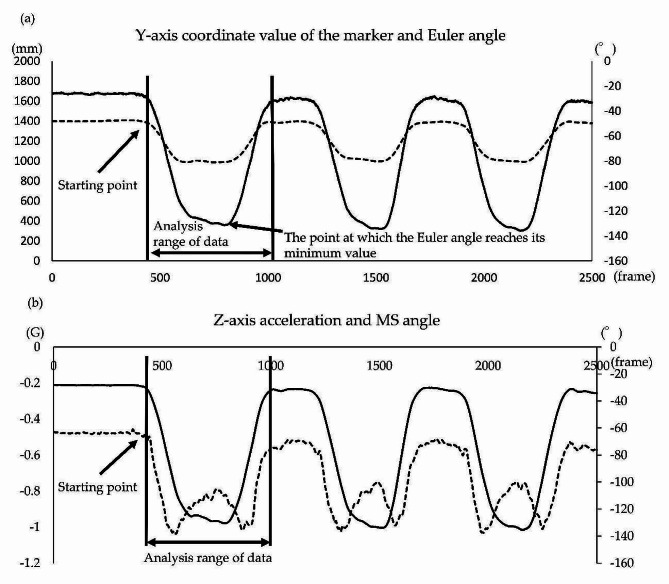
Analysis procedure of the Euler and MS angle for trunk forward bending. Analysis procedure for the Euler and MS angles during trunk forward bending. (**a**) Y-axis coordinate values (dotted lines) of the marker and Euler angles (solid lines) obtained using the three-dimensional motion analysis system. The horizontal, left vertical, and right vertical axes represent the frame number, Y-axis coordinate values of the markers, and angles, respectively. (**b**) Z-axis acceleration values (dotted lines) and MS angles (solid lines) obtained using the motion sensor. The horizontal, left vertical, and right vertical axes represent the frame number, Z-axis acceleration values, and angles, respectively. MS = motion sensor

In the 3DMA system, the initiation of motion was defined as the time point at which the value of the infrared marker located on the head side of the T1 motion sensor (Fig. 2) exceeded the value obtained by adding three times the standard deviation (SD) to the average value of the Y-axis coordinate for 1 s before the initiation of motion. The analysis range was defined as one cycle until the coordinate values of the marker returned to the values at the initiation of motion (Fig. 4-a) [32]. Furthermore, in the motion sensor, the initiation of motion was defined as the time point at which the value of the T1 motion sensor (Fig. 2) exceeded the value obtained by adding three times the SD to the average value of the Z-axis acceleration for 1 s before the initiation of motion. The analysis range was defined as one cycle until the acceleration values of the sensor returned to the values at the initiation of motion (Fig. 4-b) [32].

#### Analysis of trunk backward bending

**Fig. 5:**
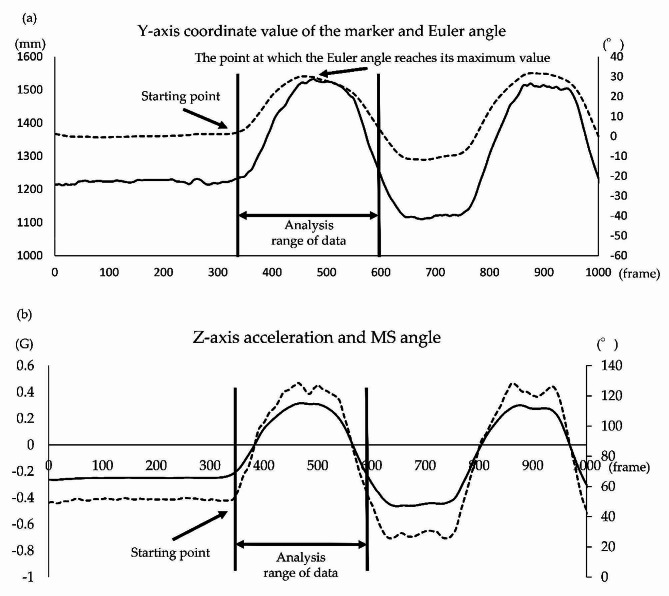
Analysis procedure of the Euler and MS angles for trunk backward bending. Analysis procedure for the Euler and MS angles during trunk backward bending. (**a**) Y-axis coordinate values (dotted lines) of the marker and Euler angles (solid lines) obtained using the three-dimensional motion analysis system. The horizontal, left vertical, and right vertical axes represent the frame number, Y-axis coordinate values of the markers, and angles, respectively. (**b**) Z-axis acceleration values (dotted lines) and MS angles (solid lines) obtained using the motion sensor. The horizontal, left vertical, and right vertical axes represent the frame number, Z-axis acceleration values, and angles, respectively. MS = motion sensor

In the 3DMA system, the initiation of motion was defined as the time point at which the value of the infrared marker on the head side of the T1 motion sensor (Fig. 2) exceeded the value obtained by adding three times the SD to the average value of the Y-axis coordinate for 1 s before the initiation of motion. The analysis range was defined as one cycle until the coordinate values of the marker returned to the values at the initiation of motion (Fig. 5-a) [32]. Furthermore, in the motion sensor, the initiation of motion was defined as the time point at which the T1 motion sensor (Fig. 2) exceeded the value obtained by adding three times the SD to the average value of the Z-axis acceleration for 1 s before the initiation of motion. The analysis range was defined as one cycle until the acceleration values of the sensor returned to the values at the initiation of motion (Fig. 5-b) [32].

The same methodology was employed in the trunk backward bending analysis. Motion initiation was defined by the placement of the infrared marker on the head side of the T1 motion sensor (Fig. 2) surpassing three times the SD added to the average Y-axis coordinate value over the preceding second. The analysis spanned one cycle until the marker’s coordinates returned to their initial values (Fig. 5-a) [32]. Furthermore, motion initiation in the sensor was determined using the T1 motion sensor (Fig. 2) surpassing three times the SD added to the average Z-axis acceleration value over the preceding second. The analysis covered one cycle until the sensor’s acceleration values returned to their initial state (Fig. 5-b) [32].

### Statistical analysis

R version 4.2.1 was used for the analysis. The velocities at the comfortable and maximum speeds were compared using the paired *t*-test. The agreement of angles in the ROM was assessed using the intraclass correlation coefficient (ICC) (2,3) of the Euler angles at the position of the minimum Euler angle during trunk forward bending (Fig. 4) and the maximum Euler angle during trunk backward bending (Fig. 5). The strength of agreement was categorized as almost perfect (0.81–1.0), substantial (0.61–0.80), moderate (0.41–0.60), fair (0.21–0.40), and slight (0.0–0.20) [33]. The error between the Euler and MS angles was validated using the mean absolute error (MAE). The interpretation of absolute error (AE) was classified as follows: ≤2°, good accuracy; 2° < AE ≤ 5°, acceptable accuracy; 5° < AE ≤ 10°, tolerable accuracy; and > 10°, unacceptable accuracy [34]. The congruence of waveforms for the Euler and MS angles was examined using the coefficient of multiple correlation (CMC) [35]. The strength of congruence in CMC was categorized as moderate (0.65–0.75), good (0.75–0.85), very good (0.85–0.95), and excellent (0.95–1.00) [36]. Sample size calculations were performed using G*Power 3.1.9.7 (Heinrich Heine University, Düsseldorf). The significance level was set at α = 0.05 and the statistical power at 1 − β = 0.8. We used the average correlation coefficient (*r* = 0.694) between the motion sensor and optical motion analysis device, which was utilized to verify the reliability of LEOMO motion sensor in a previous study [28]. Consequently, the recommended number of participants was *≥* 11.

## Results

### Angular velocity of each motion sensor during trunk forward bending

The angular velocity of trunk forward bending at a comfortable speed was − 60.5°/s ± 14.2°/s to − 104.1°/s ± 22.4°/s. In contrast, the angular velocity of trunk forward bending at the maximum speed was − 137.3°/s ± 49.9°/s to − 200.6°/s ± 37.9°/s. The angular velocities obtained from each motion sensor at the maximum speed were significantly higher than those at the comfortable speed (Table 1).

**Table 1:** Angular velocity obtained from each motion sensor for trunk forward and backward bending

	Trunk forward bending				Trunk backward bending			
	CoSp (°)	MaSp (°)	p Value	Effect size (r)	CoSp (°)	MaSp (°)	p Value	Effect size (r)
T1	-104.1 ± 22.4	-200.3 ± 36.2	< 0.01	0.90	66.3 ± 20.1	116.4 ± 42.1	< 0.01	0.82
T7	-103.9 ± 23.2	-200.6 ± 37.9	< 0.01	0.92	53.8 ± 17.1	96.6 ± 30.5	< 0.01	0.87
L1	-90.4 ± 22.4	-174.6 ± 39.5	< 0.01	0.92	43.7 ± 15.8	75.2 ± 20.6	< 0.01	0.88
L3	-75.0 ± 20.9	-150.4 ± 36.9	< 0.01	0.88	44.1 ± 34.1	72.1 ± 33.0	< 0.01	0.88
S2	-66.9 ± 30.4	-139.6 ± 53.7	< 0.01	0.88	36.0 ± 13.6	56.7 ± 16.4	< 0.01	0.89

Legend: CoSp = comfortable speed; MaSp = maximum speed

### Angular velocity of each motion sensor during trunk backward bending

The angular velocity of trunk forward bending at a comfortable speed was 36.0°/s ± 13.6°/s–66.3°/s ± 20.1°/s. In contrast, the angular velocity of trunk forward bending at the maximum speed was 56.7°/s ± 16.4°/s–116.4°/s ± 42.1°/s. The angular velocities obtained from each motion sensor at the maximum speed were significantly higher than those at the comfortable speed (Table 1).

### Validity of ROM during trunk forward bending

The agreement between the Euler and MS angles at a comfortable speed had a high agreement, with ICC (2,3) exceeding 0.9 for all segments. The angular errors between the Euler and MS angles were assessed using MAE, resulting in angles of 3.1° at T1, 2.7° at T7, 1.8° at L1, 1.1° at L3, and 1.5° at S2. Similarly, at the maximum speed, the agreement of the angle between the Euler and MS angles exhibited a high agreement, with ICC (2,3) exceeding 0.9 for all segments. The angular errors between the Euler and MS angles assessed using MAE were 2.6° at T1, 4.4° at T7, 3.9° at L1, 3.1° at L3, and 3.3° at S2 (Table 2).

**Table 2:** Agreement between Euler and MS angles in the range of motion of trunk forward and backward bending

		CoSp						MaSp				
		T1	T7	L1	L3	S2		T1	T7	L1	L3	S2
Trunk forward bending	ICC(2,3)	0.97	0.99	0.99	0.99	0.99		0.98	0.96	0.95	0.98	0.97
	MAE(°)	3.10	2.70	1.80	1.10	1.50		2.60	4.40	3.90	3.10	3.30
Trunk backward bending	ICC(2,3)	0.98	0.97	0.97	0.97	0.97		0.98	0.98	0.93	0.98	0.97
	MAE(°)	2.2	2.1	1.6	2.0	1.8		2.5	2.4	2.5	2.0	2.0

Legend: CoSp = comfortable speed; MaSp = maximum speed; ICC = intraclass correlation coefficients; MS = motion sensor; MAE = Mean absolute error

**Fig. 6:**
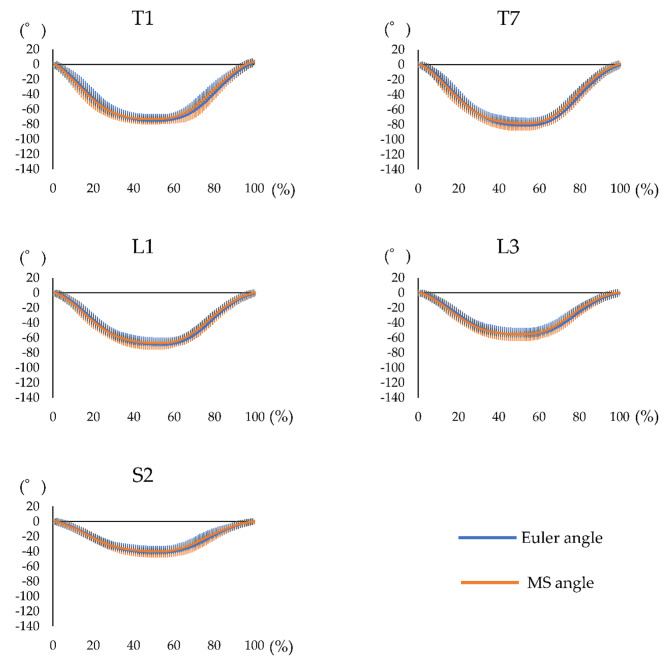
Waveform of each motion sensor for trunk forward bending at the comfortable speed. Waveform of the measurement performed using both systems, including the Euler angle obtained using the three-dimensional motion analysis system and MS angle obtained using the motion sensor, during trunk forward bending at the comfortable speed. The horizontal axis represents the frame number, and the left vertical axis represents the angle. The blue line represents the Euler angle, and the orange line represents the MS angle. MS = motion sensor

**Fig. 7:**
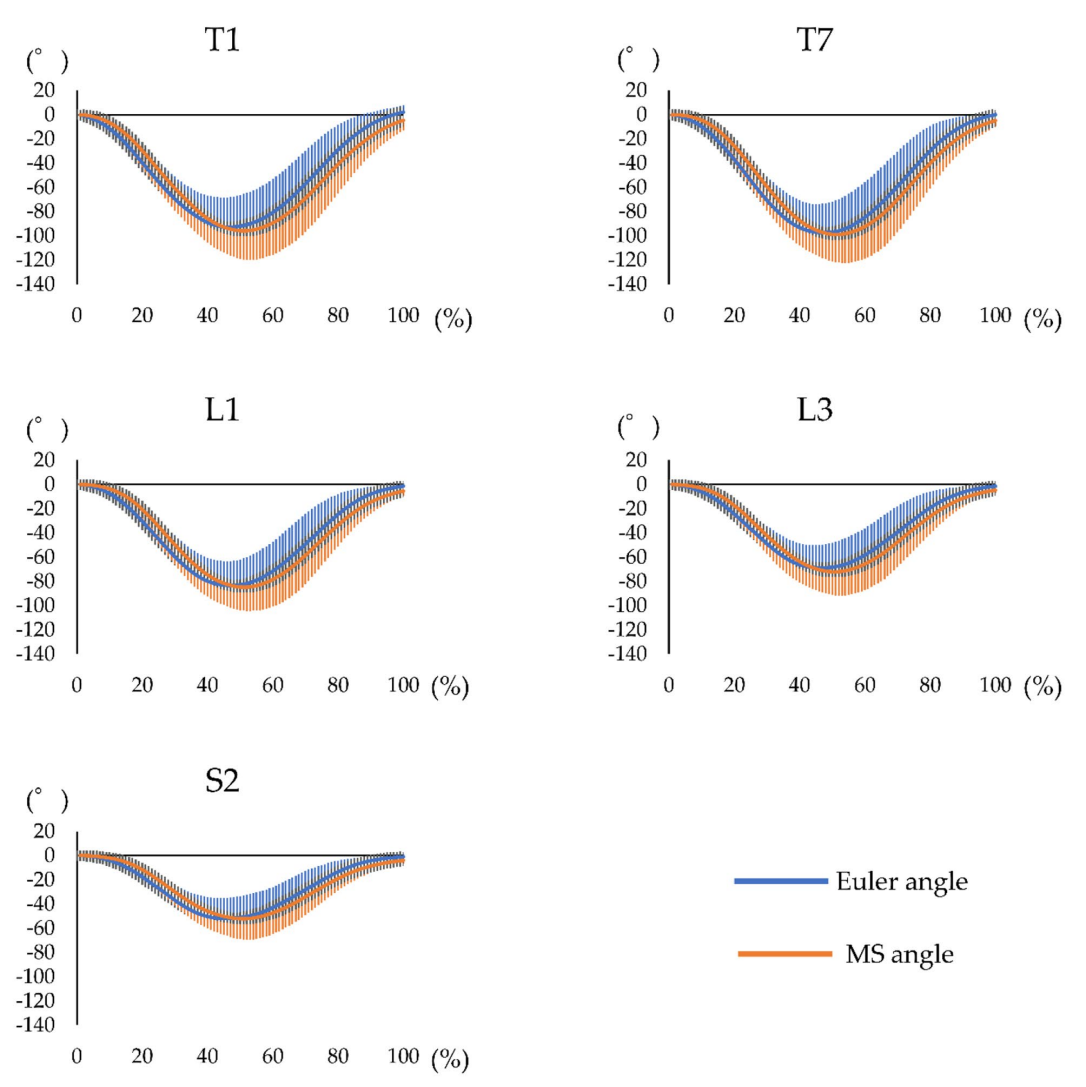
Waveform of each motion sensor for trunk forward bending at the maximum speed. Waveform of the measurement performed using both systems, including the Euler angle obtained using the three-dimensional motion analysis system and MS angle obtained using the motion sensor, during trunk forward bending at the maximum speed. The horizontal axis represents the frame number, and the left vertical axis represents the angle. The blue line represents the Euler angle, and the orange line represents the MS angle. MS = motion sensor

The waveform agreement of the Euler and MS angles at the comfortable speed had a high agreement (CMC > 0.9) for all segments (Fig. 6; Table 3). Similarly, at the maximum speed, the waveform agreement of the Euler and MS angles had a high agreement (CMC > 0.9) for all segments (Fig. 7; Table 3).

**Table 3:** Agreement between Euler and MS angles in the waveform of trunk forward and backward bending

	CoSp (CMC)						MaSp (CMC)				
	T1	T7	L1	L3	S2		T1	T7	L1	L3	S2
Trunk forward bending	0.99	0.99	0.99	0.99	0.99		0.98	0.99	0.99	0.99	0.98
Trunk backward bending	0.99	0.98	0.99	0.97	0.96		0.99	0.99	0.98	0.99	0.97

Legend: CMC = coefficient of multiple correlation; CoSp = comfortable speed; MaSp = maximum speed

### Validity of ROM during trunk backward bending

The agreement between the Euler and MS angles at a comfortable speed had a high agreement, with ICC (2,3) exceeding 0.9 for all segments. The angular errors between the Euler and MS angles were assessed using MAE, resulting in angles of 2.2° at T1, 2.1° at T7, 1.6° at L1, 2.0° at L3, and 2.0° at S2. Similarly, at the maximum speed, the agreement between the Euler and MS angles exhibited a high agreement, with ICC (2,3) exceeding 0.9 for all segments. The angular errors between the Euler and MS angles assessed using MAE were 2.5° at T1, 2.4° at T7, 2.5° at L1, 2.0° at L3, and 2.0° at S2 (Table 2).

**Fig. 8:**
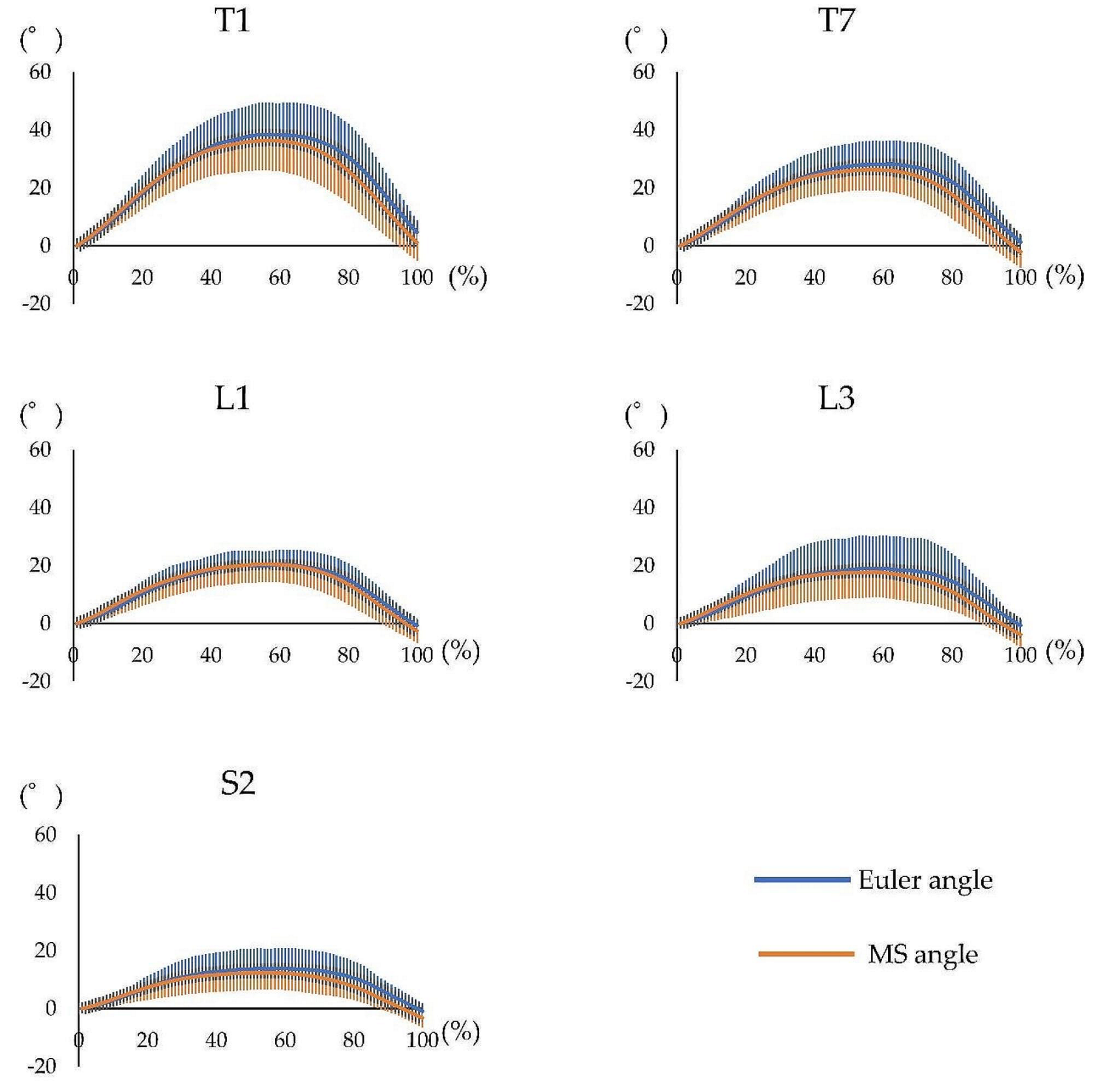
Waveform of each motion sensor for trunk backward bending at the comfortable speed. Waveform of the measurement performed using both systems, including the Euler angle obtained using the three-dimensional motion analysis system and MS angle obtained using the motion sensor, during trunk backward bending at the comfortable speed. The horizontal axis represents the frame number, and the left vertical axis represents the angle. The blue line represents the Euler angle, and the orange line represents the MS angle. MS = motion sensor

**Fig. 9:**
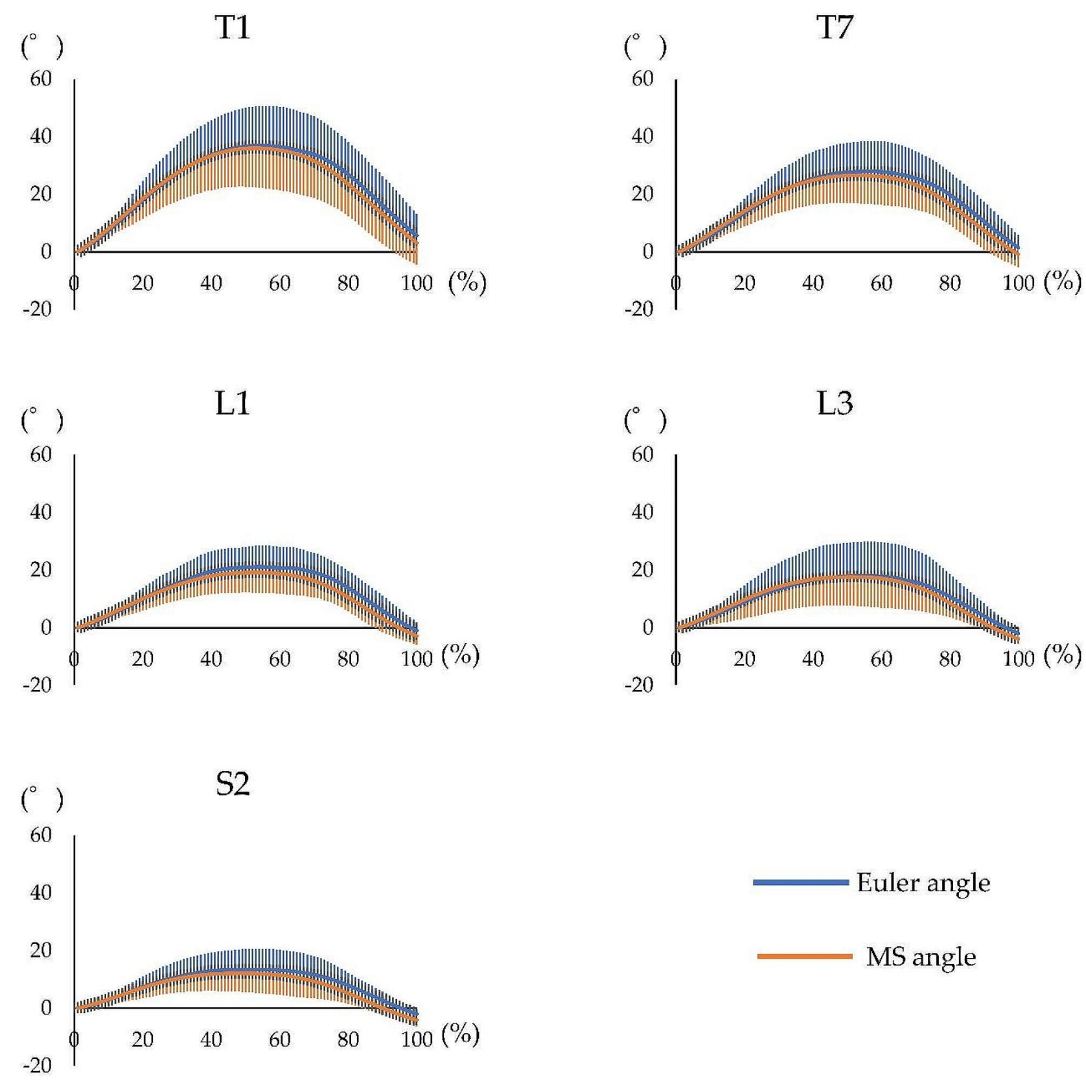
Waveform of each motion sensor for trunk backward bending at the maximum speed. Waveform of the measurement performed using both systems, including the Euler angle obtained using the three-dimensional motion analysis system and MS angle obtained using the motion sensor, during trunk backward bending at the maximum speed. The horizontal axis represents the frame number, and the left vertical axis represents the angle. The blue line represents the Euler angle, and the orange line represents the MS angle. MS = motion sensor

The waveform agreement of the Euler and MS angles at the comfortable speed had a high agreement (CMC > 0.9) for all segments (Fig. 8; Table 3). Similarly, at the maximum speed, the waveform agreement of the Euler and MS angles presented with a high agreement (CMC > 0.9) for all segments (Fig. 9; Table 3).

## Discussion

This study assessed the validity of the estimated angles obtained using motion sensors during trunk forward and backward bending. The results revealed high ROM and waveform validity during trunk forward and backward bending at both comfortable and maximum speeds.

The validity of ROM during trunk forward and backward bending was assessed using ICC (2,3). Results revealed values exceeding 0.9 (perfect) at both the comfortable and maximum speeds. In addition, the MAE of the MS angles relative to the Euler angles was calculated. The MAE ranges up to 4.4° for each motion sensor. In a previous study assessing the validity of motion sensors during trunk movements, the reported errors were 1.82° ± 1.00° for trunk forward bending and 0.71° ± 0.34° for trunk backward bending [37]. An MAE value of < 5° indicated acceptable accuracy [34]. Clinical evaluations using goniometers typically consider measurements in 5° increments as a standard [38]. Therefore, the MAEs in this study were clinically acceptable.

Quantitative assessment of human movements during rehabilitation is important for understanding impairments and developing effective treatment strategies. The evaluation methods included ROM assessment and acquisition of time-series data to evaluate movement patterns (waveforms). The traditional methods for assessing ROM include tools such as goniometers, inclinometers, kyphometers, and rulers [5–7]. However, these methods only capture the magnitude of changes from the start to the end of the movement, leaving the trajectory unknown. Previously, optical systems have been used to evaluate spinal movement patterns during trunk forward and backward bending [11, 39]. Although the 3DMA system has high accuracy, its clinical use is limited by space requirements and high costs [40]. The motion sensors used in this study, which are affordable, lightweight, and compact and can perform outdoor measurements, have fewer disadvantages than optical systems. The waveforms obtained using motion sensors during trunk forward and backward bending had an excellent congruence (CMC > 0.95) compared with those obtained using the Euler angles in this study. Thus, the use of motion sensors in clinical settings outside the laboratory is promising.

A recent study developed video-based artificial intelligence motion analysis systems [41]. Although these systems had high accuracy in measuring limb movements, analyzing spinal movements during forward bending of the thoracic and lumbar spine is challenging [14–16]. The motion sensors used in this study can be attached to any location on the spine to calculate angles. Therefore, in clinical settings, the integration of motion sensors and artificial intelligence motion analysis systems can facilitate a comprehensive movement analysis, covering the whole body.

The current study had several limitations. First, only the angle in the X-axis rotation was evaluated. Hence, angles in other axis rotations remained unclear. Second, the potential influence of soft tissues caused by attaching motion sensors on the skin was not examined. Finally, this study verified the validity of the absolute angles obtained using each motion sensor. Therefore, the relative angles between the sensors could not be validated. Next, we plan to select key areas as evaluation indicators and examine the validity of the sensors.

## Conclusions

This study compared the validity of the estimated angle data obtained using a motion sensor and the 3DMA system. The results showed that the motion sensor had high validity. Hence, it can be a potential objective evaluation tool during rehabilitation in clinical settings. Nevertheless, further research should be performed to assess the use of this technology for assessing and treating movement patterns related to spinal diseases and establish its application in clinical settings.

### Electronic supplementary material

Below is the link to the electronic supplementary material.

Supplementary Material 1

## Data Availability

All data generated or analyzed during this study are included in this published article.

## Abbreviations

3DMA: Three-dimensional motion analysis
AE: Absolute error
CMC: Coefficient of multiple correlation
CoSp: Comfortable speed
ICC: Intraclass correlation coefficient
L1: First lumbar spinous process
L3: Third lumbar spinous process
MAE: Mean absolute error
MaSp: Maximum speed
MS: Motion sensor
S2: Second sacrum spinous process
SD: Standard deviation
T1: First thoracic spinous process
T7: Seventh thoracic spinous process
